# Evaluating a prototype digital mental health literacy intervention for children and young people aged 11–15 in Java, Indonesia: a mixed methods, multi-site case study evaluation

**DOI:** 10.1186/s13034-023-00608-9

**Published:** 2023-06-26

**Authors:** Helen Brooks, Irmansyah Irmansyah, Armaji Kamaludi Syarif, Rebecca Pedley, Laoise Renwick, Atik Puji Rahayu, Christa Manik, Benny Prawira, Mark Hann, Helen Brierley, Karina Lovell, Penny Bee

**Affiliations:** 1 Division of Nursing, Midwifery and Social Work, School of Health Sciences, Manchester Academic Health Science Centre, University of Manchester, Jean McFarlane Building, Oxford Road, Manchester, UK; 2 National Research and Innovation Agency, Jakarta, Indonesia; 3 Ministry of Health, Jakarta, Indonesia; 4 Marzoeki Mahdi Hospital, Bogor, Indonesia; 5 Independent Psychological Researcher, Jakarta, Indonesia; 6 Centre for Primary Care and Health Services Research, University of Manchester, Manchester, UK; 7 Manchester Mental Health NHS Foundation Trust, Manchester, UK

**Keywords:** Mental health, Digital applications, Mental health literacy, Self-management, Co-design, Anxiety, Depression, Indonesia

## Abstract

**Background:**

The Improving Mental Health Literacy Among Children and Young People in Indonesia (IMPeTUs) intervention is a co-produced, evidence-based digital intervention designed to improve anxiety and depression focused mental health literacy and self-management among people aged 11–15 in Java, Indonesia. This study aimed to evaluate the usability, feasibility and preliminary impact of our intervention.

**Methods:**

Mixed methods, multi-site case studies based on a theory of change. Pre-and post-assessments of a range of outcomes and qualitative interviews/focus groups with children and young people (CYP), parents and facilitators. The intervention was implemented in 8 health, school and community sites across Java, Indonesia (Megelang, Jakarta and Bogor).

Quantitative data designed to understand the impact of and feasibility of evaluating the intervention collected from 78 CYP who used the intervention were analysed descriptively. Qualitative data from interviews and focus groups collected from 56 CYP, 49 parents/caregivers and 18 facilitators were analysed using framework analysis.

**Results:**

Qualitative data analysis indicated high levels of usability and acceptability for the interface aesthetic, personalisation, message presentation and navigation. Participants reported minimal burden and no negative outcomes associated with the intervention. CYP, parents and facilitators identified a range of direct and spill over effects of interventions engagement, some of which were not anticipated at study outset. Quantitative data highlighted the feasibility of intervention evaluation, with high levels of recruitment and retention across study time points. Minimal changes were identified in outcomes pre-to-post intervention, which may in part be due to a lack of scale relevance and/or sensitivity to the intervention mechanisms indicated in the qualitative data.

**Conclusions:**

Digital mental health literacy applications are potentially an acceptable and feasible way to prevent burdens of common mental health problems amongst CYP in Indonesia. Our intervention and evaluative processes will be further refined prior to definitive evaluation.

**Supplementary Information:**

The online version contains supplementary material available at 10.1186/s13034-023-00608-9.

## Introduction

Mental health difficulties are significant contributors to the global burden of disease and affect between 10–20% of children and young people (CYP) across the world [1]. 50% of mental health problems are established by the age of 14 [2]. Depression, in particular, is considered a leading cause of mental health disability, estimated to affect between 4 and 8% of adolescents globally each year [3]. A recent meta-analysis estimated global prevalence of anxiety amongst CYP to be 20.5% which is considered to have doubled since the Covid-19 pandemic [4].

Evidence has demonstrated an individual’s risk of experiencing commnon mental health problems increases significantly after puberty and that approximately two-thirds of CYP with depression will have a recurrent episode within five years [5, 6]. The long-term negative effects of childhood mental health difficulties are well documented. Experiencing mental health difficulties in childhood is linked to co-morbid physical health problems, more serious and persistent mental health episodes later in life and an increased risk of suicide [7, 8].

Indonesia, an archipelago in Southeast Asia, meets World Bank criteria for a lower-middle income country (LMIC). Recent evidence suggests that nearly 50% of high school students living in Indonesia experience depressive symptoms and suicidal ideation has a 12-month prevalence of 6.8% [9]. The United Nations Sustainable Development plan sets down targets for reducing mortality by 33% by 2020, to be driven by the prevention and treatment of non-communicable diseases and the promotion of mental health and wellbeing [10, 11]. Like most LMICs, treatment gaps in Indonesia are significant and often exceed 75%, highlighting an urgent need to scale up effective and acceptable support. Mental health is a national priority in Indonesia; however, recent decentralisation of mental health care means community provision is still in the early stages of development and significant challenges remain [12].

Long standing treatment demands have been exacerbated by recent global events. For example, Covid-19 has significantly impacted on the mental health of CYP. There has been a myriad of government/policy, third sector and academic reports published signalling the concern for mental health and wellbeing among adults and CYP [13]. While the focus on transforming services to meet the needs of CYP with poor mental health is a global policy imperative [14], the pandemic has highlighted and exacerbated the gap between the needs of CYP and the solutions on offer. The current pandemic presents an opportunity to develop preventative solutions which are cost-effective in the short- and longer term. Such solutions have the potential to support CYP today to become healthier future generations of adults [15, 16].

Mental health literacy (MHL), the ‘knowledge and belief about mental disorders which aid their recognition, management or prevention’ is a promising development in global mental health research [17]. The MHL framework originally developed by Jorm and colleagues comprises of a range of skills clustered within four components:

- the ability to identify mental health difficulties
- recognising the causes of mental health difficulties
- understanding of effective help-seeking
- perceptions of the factors that impact on help-seeking [17].

This original conceptualisation has since been expanded to include perceptions of positive mental health and wellbeing, self-efficacy and stigma [18–20].

Mental health literacy skills have consistently been shown to be associated with improved health outcomes and social determinants of health. The available evidence suggests improved recognition and knowledge about mental illness and appropriate support can improve help-seeking in CYP [21]. This is important given that studies show as little as one third of adolescents seek appropriate treatment for mental health problems. Timely and appropriate help seeking in early adolescence is critical for recovery, and for reducing the likelihood of mental disorder in later adolescence and life [22]. Poorer mental health literacy has been associated with increasing mental illness among CYP [23].

This paper reports on the feasibility evaluation of the IMPeTuS intervention which was co-designed to improve mental health literacy amongst CYP aged 11–15 in Java, Indonesia [24].

### The IMPEtUS intervention

Stepped care prevention and treatment approaches, where psychological interventions are delivered according to need, are considered an optimal solution to reduce the burden on health systems [25]. Our solution is a mental health intervention designed to improve self-management of mental health and timely intervention through improved mental health literacy, recognition and effective help-seeking [26].

The IMPeTUs intervention was co-designed with CYP, parents, professionals and games designers in Java, Indonesia [26]. Systematic reviews were undertaken in preparation for this co-production which demonstrated a dearth of culturally sensitive MHL interventions which were appropriate for use in Indonesia, which required the co-design of a novel intervention [26–28]. Intervention development adhered to the recommendations outlined in the Medical Research Council’s guidelines for the development of complex interventions [29] and links to the WHO strategy for improving resources in schools to support CYP mental health [11].

Drawing on principles of Experience Based Co-design [30], our intervention co-production comprised three phases. The first phase involved an initial needs assessment in the form of systematic reviews [26–28] and in-depth qualitative data obtained from CYP, parents, and health and education professionals [31, 32]. These data informed phase 2 which centred on a stakeholder consensus exercise and 3-day co-production workshop with 50 attendees (CYP, parents, health and education professionals, researchers and games designers). This decided a design brief for the format and provisional content of the intervention along with implementation strategies. In the third and final phase the games designers worked closely with our patient and public involvement group in ongoing group work to ensure the intervention was developed and refined in line with the original co-produced brief [26].

**Fig. 1:**
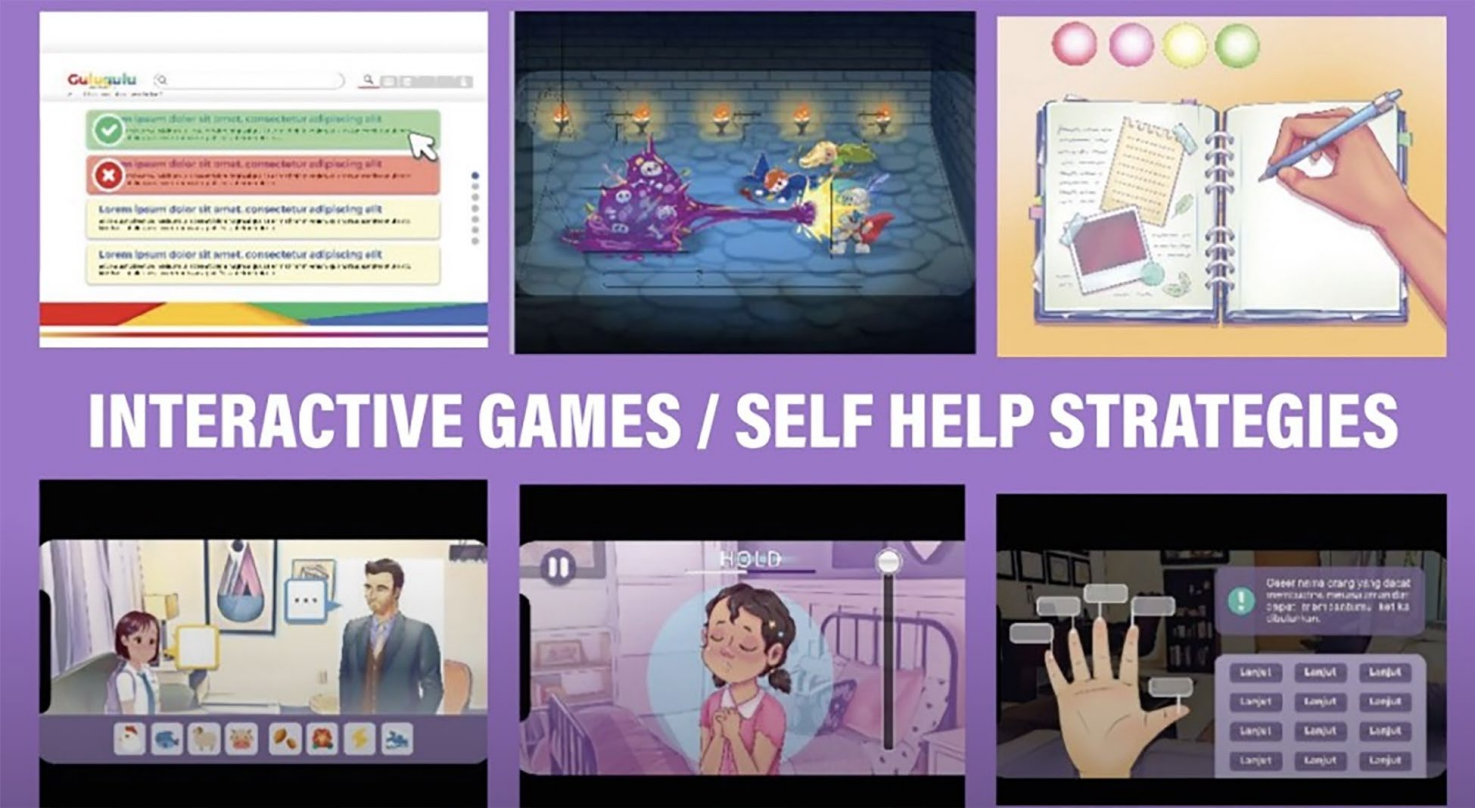
Example artwork from the IMPeTUs intervention (originally published in [24]

**Fig. 2:**
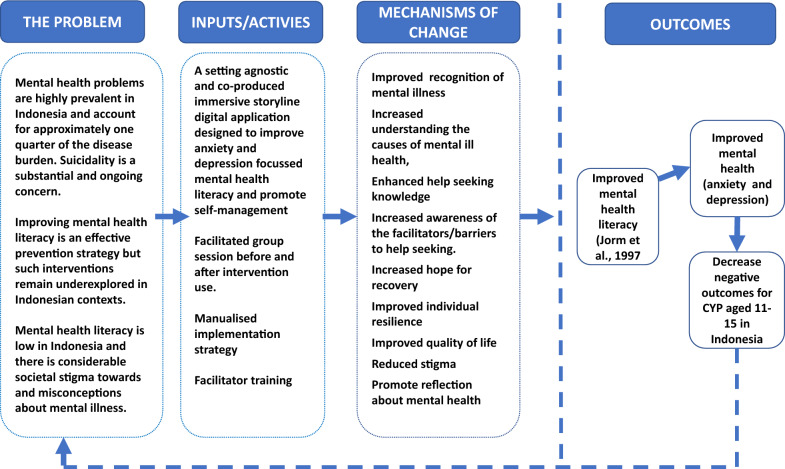
Preliminary theory of change originally published in [26]

The IMPeTUs intervention includes a digital application and facilitated group discussion sessions for CYP before and after engagement with the intervention. Group discussions were considered necessary to enable CYP to be suitably oriented to the intervention and to provide a space in which CYP could discuss issues raised with the game and discuss any issues which arose during game play. The digital application takes the form of an immersive storyline design in which CYP take on the role of a central, customisable character and navigate the character through a series of mental health related challenges. There is one chapter of the game which focuses on depression and one chapter that focuses on anxiety. The immersive storyline design is broken up with interactive games and exercises to promote engagement with the applications. Screen art from the digital application can be found in Fig. 1 and a preliminary theory of change developed by the authors can be found in Fig. 2.

### Aims

In line with the WHO guidelines for the evaluation of prototype mHealth interventions, our case studies aimed to understand the usability, feasibility and preliminary imact of our prototype intervention [33]. In the WHO guidelines, efficacy relates to the extent to which an intervention is thought to bring about intended outcomes in a research setting:

- To qualitatively examine the usability, feasibility and impact of our co-produced intervention.
- To quantitatively explore the preliminary impact of our co-produced intervention on a range of user-focussed outcome measures.
- To explore the feasibility of evaluating our co-produced intervention.

## Methods

### Study design

The study was designed to evaluate a prototype digital mental health literacy and depression and anxiety focussed self-management intervention. Our evaluation was informed by the User Focussed Evaluation approach developed by Patton (1997) [34]. This approach is based on a fundamental principle that an evaluation should be assessed on its perceived utility to its intended beneficiaries. Our previous endeavours to include relevant stakeholders in the design of the intervention are described previously [26]. We also worked closely with patient and public involvement groups comprised of CYP and adults with lived experience of mental health difficulties in Indonesia to design the initial theory of change and to design evaluation processes.

In order to address the study aims, a collective mixed-methods case study design was employed which incorporated multiple cases undertaken concurrently [35]. With equal weight given to both types of study design [36], quantitative and qualitative data were used to answer different elements of the study aims. Therefore, it was not necessary to integrate the two types of study data; however, synergies between the findings are considered in the discussion [37].

### Setting

Our case study evaluations were undertaken in three geographical areas of Java, Indonesia. These were Jakarta, Magelang and Bogor. Sites were selected on their differences in culture, urbanisation and level of health service provision. These sites also had specific child and adolescent mental health service provision which was considered useful in terms of supporting the research and evaluating the prototype intervention [24]. We aimed to recruit one school, one community organisation and one health service setting in each geographical site resulting in a total of 9 case study evaluations. However, the evaluative period coincided with the global Covid-19 pandemic and the health service site in Jakarta did not have capacity to undertake the case study evaluation due to ongoing pressures relating to coronavirus. This resulted in 8 case study evaluations being completed.

## Participants

### Children and young people

CYP were eligible to take part if they were aged 11–15 and in contact with one of the recruitment sites across our 8 case study evaluation locations. Participants were required to provide assent and parental consent prior to participation in study activities. A total of 78 CYP were recruited to the quantitative element, 56 also took part in an interview or focus group. Further demographic information can be found in Table 1.

**Table 1:** Demographic data

Children and Young People^a^	% (n)
Gender	
Male	51.3% (n = 39)
Female	48.7% (n = 37)
Age	
11	23.6% (n = 18)
12	17.1% (n = 13)
13	15.8% (n = 12)
14	30.3% (n = 23)
15	13.2% (n = 10)
Currently experiencing mental health difficulties	
Yes	25% (n = 19)
No	71.1% (n = 54)
Don’t know	3.9% (n = 3)
Type of mental health problem currently experienced	
Depression	5/19
Anxiety	13/19
Other	5/19
Duration of illness for those currently experiencing mental health problems	
Mean	14 months
Range	6–36 months
For those currently experiencing mental health problems, help had been sought from:	
Family	17/19
Friends	4/19
Health professionals	15/19
Teachers	3/19
Third sector	0/19
App usage (number of times the intervention was engaged with)^b^	
Mean	5
Range	1–15
Parents/family members^c^	
Gender	
Male	14.5% (n = 11)
Female	85.5% (n = 65)
Age	
Mean	43 years
Range	32–56 years

^a^Demographic data was collected from 76/78 CYP

^b^Parent reported

^c^Demographic data was collected from 76/78 parents who consented for their CYP to take part in the evaluation

### Parents/caregivers

Caregivers were eligible to take part in the study if they had a CYP they provided care for aged 11–15 who had agreed to take part in the prototype evaluation. Caregivers were required to give written and informed consent to take part in study activities. A total of 78 parents/caregivers provided consent for they CYP to take part in the study and 49 provided informed consent and took part in an interview or focus group. Further demographic details are provided in Table 1.

### Facilitators/professionals

All facilitators who were involved in implementing the IMPeTUs intervention were invited to participate in the case study evaluations. Other professionals were eligible to participate in the case study evaluations if they were working at the sites that CYP taking part in the case study evaluations attended and had some experience of the intervention. A total of 18 facilitators/professionals were recruited to the study. Facilitators were professionals working within study sites.

### Procedures

CYP were recruited to the study through direct invitation from the organisations they attended (school, health service or community venue) in each case study site. Staff within participating organisations identified CYP aged 11–15 who were eligible to take part in the evaluation. Study information packs containing an invitation letter, information sheets for both CYP and parents, a consent form and contact details form with return envelope were distributed to all eligible CYP. These were distributed to eligible participants on behalf of the research team by post, email or in person during routine contacts. CYP received a age-appropriate version of the information sheet. Parents/caregivers completed and returned consent forms and completed contact details if their child was happy to take part and had given their verbal assent.

Parents/caregivers of those children who agreed to take part in the study also received a separate invitation letter, participant information sheet, consent form and contact details form in order to invite them to take part in an individual interview or focus group to discuss their child’s experience of using the prototype intervention.

Invitation packs containing an invitation letter, participant information sheet, consent to contact form and consent form were sent by post or email directly from the research team to eligible facilitators/professionals.

Those interested in taking part returned the consent form/consent to contact form and contact details form to the research team who contacted them to discuss the study further, answer any questions and to facilitate access to an upcoming focus group or arrange a one-to-one interview, depending on individual preference. All participants (CYP, parents and professionals) were offered a small cash payment in recognition of their time taking part.

### Quantitative data

**Fig. 3:**
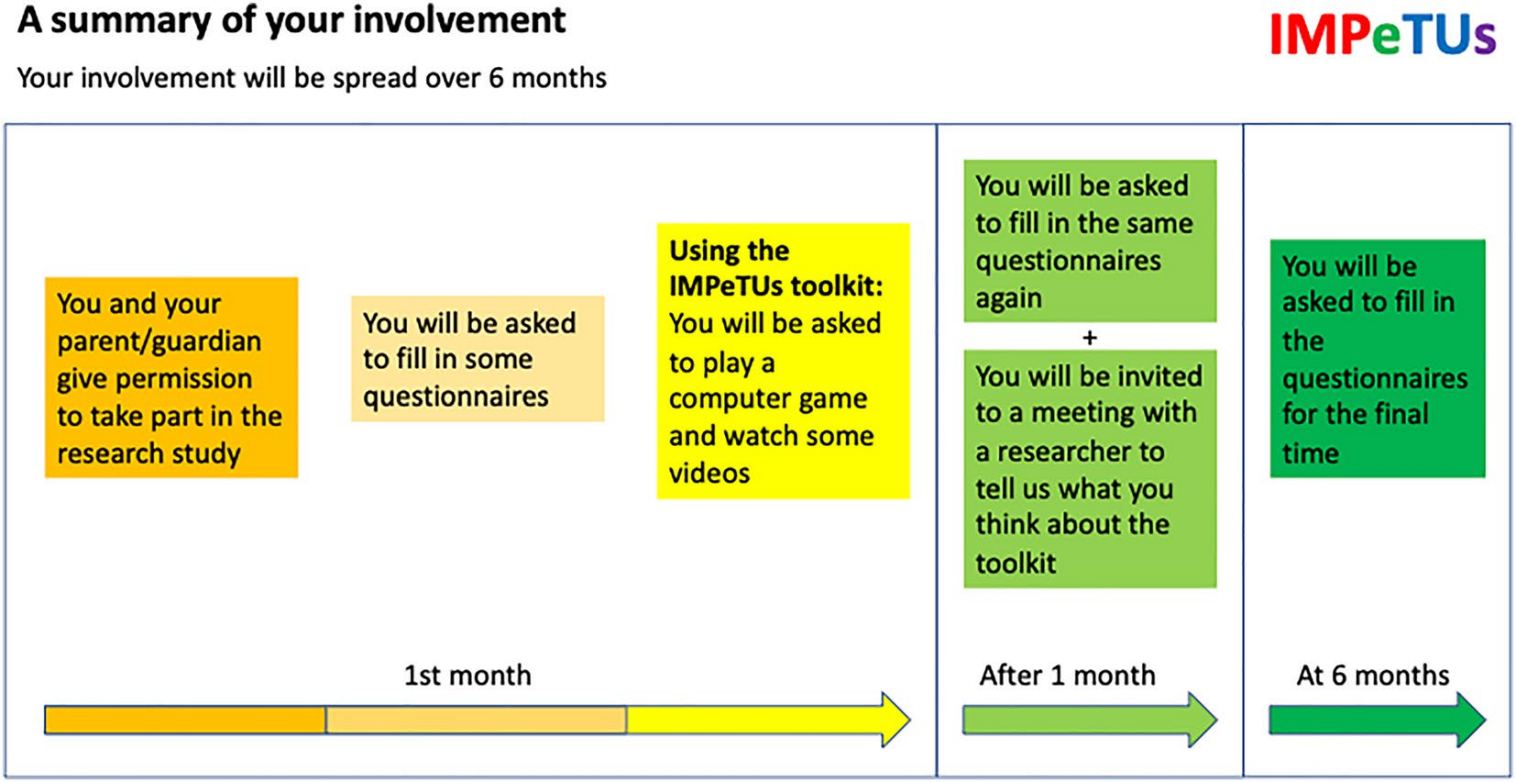
Timeline of CYP involvement in the study. This diagram was included in the CYP information sheet

As shown in Fig. 3, CYP were invited to complete a set of questionnaires at three timepoints: prior to using the game (baseline), on a second occasion after playing the game for one month (post-intervention) and a final time six months after baseline. The measures included in the pack are detailed in Table 2 and included a range of self-complete questionnaires developed for CYP which were relevant to the initial ToC and chosen by the lived experience panel.

**Table 2:** CYP measures and completion timepoints

Quantitative measure name	Additional information	Timepoints used	Score calculation
Demographic & background information questionnaire	Purpose developed for the study	Baseline	N/A
Knowledge and Attitudes to Mental Health Scales (KAMH) Pupil version^a^ [45]	Translated to Bahasa Indonesia by a member of the study team and verified by a second member of staff	Baseline, 1 month and 6 months	Total score utilised
Paediatric Quality of Life Inventory (PEDS-QL) generic core scale^a^—versions for age 13–18 years and 8–12 years [46] Bahasa Indonesia version supplied by distributor	Minor amendments made to improve the quality of Bahasa Indonesia translation. Changes verified through cognitive interviews and approved by Mapi Research Trust and the author	Baseline, 1 month and 6 months	Total score utilised in addition to the physical and psychosocial sub-scales
Zung Self-rated Anxiety scale—Bahasa Indonesia version [47]	Agreed with authors to specify a timeframe to the measure’s instructions (over the past week)	Baseline, 1 month and 6 months	Total score utilised
Reynolds Adolescent Depression Scale^b^ (RADS 2; Reynolds) [48]	Professionally translated from English to Bahasa Indonesia and back translated, in accordance with the distributor’s requirements	Baseline, 1 month and 6 months	Items summed to generate total score
The Family Adaptability and Cohesion Scale^b^ (FACES IV)—Family Communication scale and Family satisfaction scale [49] Bahasa Indonesia version supplied by distributor	Distributor agreed for 2 scales to be extracted from Bahasa Indonesia translation	Baseline, 1 month and 6 months	Total scores for family communication and family satisfaction sub-scales utilised
Questionnaire collecting number of times using the game	Purpose developed for the study	1 month	N/A

^a^These measures differ from those specified in the protocol [22] to ensure cultural and age appropriacy

^b^The original protocol [24] specified earlier versions of these measures (i.e. FACES II and RADS) however later versions were employed for the study as they became available

### Qualitative data

After playing the game over approximately a one-month period, CYP took part in a one-time qualitative interview or focus group depending on participant preference lasting up to one-hour, guided by a topic guide exploring 56 CYP experiences and perceptions of the intervention. 49 parents of children who had tried the intervention and 18 professionals involved in the evaluation took part in a one-time interview or focus group lasting 60–90 min. Interviews/focus groups explored parental views about the intervention and perceptions of their child’s experiences whereas professional interviews explored their perceptions of the intervention based on their experience of delivering the intervention. 21/56 CYP, 2/49 parents and 18/18 facilitators took part in in-person interviews and focus groups and the rest were undertaken via zoom. Interviews/focus groups were digitally audio-recorded, transcribed verbatim and translated into English for the purposes of analysis.

## Data analysis

### Quantitative data

Scores on each of the questionnaires at each of the three time-points are summarised in Tables 4 and 5. As all of the questionnaires give a score on a continuous scale, the mean, standard deviation and range of each was calculated. To determine whether the game showed signs of preliminary efficacy (e.g., improving quality-of-life, etc.), ‘change’ scores between baseline and one-month post-intervention and baseline and six-months post-intervention were calculated and summarised as above.

Parents reported the CYP played with the intervention on average 5 times, ranging from 1–15 times.

### Qualitative data

Qualitative data were analysed using a framework approach incorporating deductive and inductive coding. Deductive coding was informed by Theoretical Framework of Acceptability (TFA) [38] and inductive coding was utilised to ensure data was captured that fell outwith this framework (Table 3).

**Table 3:** Theoretical framework of acceptability [38]

TFA component	Definition
Affective attitude	How participants feel about the intervention
Burden	How much effort participants feel that the need to put in order to engage with the intervention
Ethicality	The perceived fit between the values of the intervention and an individual's own belief system
Intervention coherence	How well participants understand the intervention and how it works
Opportunity costs	The perceived benefits, profits or values that need to be relinquished in order to engage with an intervention
Perceived effectiveness	The extent to which participants believe that the intervention will meet its intended purposes
Self-efficacy	How well participants feel they can engage with intervention activitiesHow well participants feel they can engage with intervention activities

The process of analysis followed the stages outlined by Gale et al. [39]. After transcription and anonymisation, transcripts were allocated to one of 8 coders. Each analyst familiarised themselves with the data through active reading. Next, researchers moved to charting the data in an a priori matrix which was developed using the TFA. This involved writing written summaries of the data pertaining to each TFA component in each transcript. Supporting quotes were inputted into the relevant matrix cells to support the write-up process. There were areas of the Excel sheet where additional data that fell outside the TFA framework could also be captured. Researchers were asked to keep analytical memos and document any emerging thoughts/feelings about the data as this process was undertaken.

Once all the transcripts had been analysed, two authors (HBrooks/PBee) met to verify the allocation of data to the various TFA components and to identify key issues relating to acceptability of the IMPeTUS intervention within each component. This was done collaboratively with consensus reached for each TFA component before sharing with the wider team for further verification. Each component was then written up with sufficient detail provided for each section and direct quotations provided to support interpretations.

## Results

### Quantitative results

78 CYP provided consent to take part in the study and completed baseline data collection. 76 provided demographic data. 71/78 (91%) completed assessments at one month post intervention and 70/78 (90%) at six-months post intervention. Those who dropped out were either non-contactable or declined to take part in further data collection. Where assessments were completed, they were done so in full. Thus, no data imputation for partial non-response was required. Change scores were only calculated where there was baseline and follow-up data for participants.

Table 1 shows that the average age of the 76 participants who provided data was nearly 13 years. Thirty-nine (51.3%) were male. The male participants were marginally older, primarily due to a relatively larger percentage of 14 year-olds.

Exactly one-quarter (n = 19) reported experiencing at least one mental health problem. The most commonly reported problem was anxiety (n = 13), with depression and other mental health problems being relatively less common (both reported by five participants). The median duration of the reported problems was 12 months (range = 6 to 36 months). Nearly all of those reporting a mental health problem had sought help from their family (n = 17) and/ or a health professional (n = 15). Other sources of help seeking were much less common.

Table 4 shows that the mean changes from baseline on all outcome measures were small at both one month and six months post-intervention. Table 5 outlines the average changes from baseline on all outcome measures for the 19 participants currently experiencing mental health problems vs those who were not currently experiencing mental health problems.

**Table 4:** Outcome scores for all participants across all timepoints

Outcome	Baseline	Baseline (if response at FU^*^1)	FU1	Change (FU1—B/L)	Baseline (if response at FU2)	FU2	Change (FU2—B/L)
N	78	71	71	71	70	70	70
PedSQL							
Mean (SD)	71.1 (15.4)	70.5 (15.1)	73.1 (13.3)	2.5 (10.9)	70.2 (15.0)	71.8 (14.1)	1.6 (12.2)
Median (IQR)	71.2 (63.0, 81.5)	70.7 (63.0, 79.3)	72.8 (64.1, 82.6)	0 (− 4.3, 8.7)	70.1 (63.0, 79.3)	71.2 (65.2, 81.5)	0 (− 6.5, 7.6)
Range	33.7 to 100	33.7 to 100	37.0 to 100	− 26.1 to 33.7	33.7 to 100	37.0 to 100	− 26.1 to 35.9
PedSQL Physical Health	75.0 (17.4)		76.3 (15.3)			74.4 (16.8)	
PedSQL Psycho-Social Health	69.0 (16.7)		71.4 (14.5)			70.9 (14.8)	
FACES: FamCom							
Mean (SD)	34.3 (8.5)	34.2 (8.7)	34.7 (8.4)	0.5 (4.7)	34.1 (8.7)	35.5 (8.2)	1.3 (5.3)
Median (IQR)	35.5 (28, 41)	35 (28, 41)	35 (29, 41)	0 (− 1, 3)	34.5 (28, 41)	36.5 (30, 43)	0 (− 1, 4)
Range	14 to 50	14 to 50	17 to 50	− 21 to 14	14 to 50	17 to 50	− 21 to 16
KAMH							
Mean (SD)	115.8 (14.8)	115.7 (15.0)	116.5 (17.5)	0.8 (10.3)	115.4 (15.0)	114.9 (18.0)	− 0.5 (11.7)
Median (IQR)	115 (105, 127)	115 (102, 128)	116 (101, 131)	0 (− 3, 6)	115 (102, 127)	115 (102, 130)	0 (− 4, 5)
Range	85 to 158	85 to 158	74 to 157	− 36 to 27	85 to 158	65 to 158	− 45 to 30
Anxiety							
Mean (SD)	34.9 (8.7)	35.3 (8.7)	35.1 (9.6)	− 0.3 (6.0)	35.5 (8.6)	35.3 (9.6)	− 0.2 (7.9)
Median (IQR)	34 (29, 40)	35 (31, 40)	34 (29, 39)	0 (− 1, 2)	35 (31, 40)	33 (30, 40)	0 (− 4, 4)
Range	20 to 59	20 to 59	20 to 67	− 25 to 19	20 to 59	20 to 67	− 26 to 26
Depression							
Mean (SD)	57.7 (16.4)	57.7 (16.8)	57.4 (15.3)	− 0.3 (10.3)	58.0 (16.6)	58.9 (14.3)	0.9 (12.9)
Median (IQR)	54 (45, 66)	54 (45, 66)	54 (46, 69)	0 (− 5, 5)	54 (46, 66)	58 (47, 66)	0 (− 7, 8)
Range	30 to 110	30 to 110	33 to 104	− 29 to 33	30 to 110	35 to 104	− 33 to 39

FU^*^ follow-up

**Table 5:** Outcomes for participants experiencing mental health problems (MHP) vs. no mental health problems (no MHP)

Outcome		Baseline	Baseline (if response at FU^*^1)	FU1	Change (FU1 – B/L)	Baseline (if response at FU2)	FU2	Change (FU2 – B/L)
N (MHP/no MHP)		19/53	19/49	19/49	19/49	19/48	19/48	19/47
PedSQL								
Mean (SD)	MHP	63.2 (15.2)	63.2 (15.2)	68.1 (12.8)	5.0 (15.6)	63.2 (15.2)	66.5 (13.4)	3.3 (16.8)
Median (IQR)	MHP	66.3 (50.0, 73.9)	66.3 (50.0, 73.9)	70.7 (57.6, 76.1)	3.3 (-6.5, 20.7)	66.3 (50.0, 73.9)	68.5 (62.0, 72.8)	2.2 (− 6.5, 9.8)
Mean (SD)	no MHP	74.7 (13.8)	73.6 (13.6)	75.3 (12.4)	1.7 ( 8.8)	73.3 (13.5)	74.2 (13.4)	1.0 (10.4)
Median (IQR)	no MHP	73.9 (66.3, 83.7)	72.8 (66.3, 81.5)	73.9 (67.4, 84.8)	0.0 (− 3.3, 5.4)	72.3 (66.3, 80.4)	73.9 (67.4, 84.2)	0.0 (− 6.0, 7.1)
FACES: FamCom								
Mean (SD)	MHP	34.3 (6.4)	34.3 (6.4)	35.9 (6.2)	1.6 (3.4)	34.3 (6.4)	36.5 (7.3)	2.2 (5.0)
Median (IQR)	MHP	33 (31, 41)	33 (31, 41)	36 (34, 41)	2 ( 0, 3)	33 (31, 41)	38 (30, 42)	2 (− 3, 7)
Mean (SD)	no MHP	41.0 (7.6)	40.8 (7.7)	40.2 (6.8)	− 0.6 (5.0)	40.7 (7.8)	39.0 (6.4)	− 1.7 (5.5)
Median (IQR)	no MHP	42 (37, 47)	42 (37, 47)	41 (38, 46)	0 (− 1, 0)	42 (37, 47)	39 (36, 44)	− 1 (− 5, 0)
FACES: Satis								
Mean (SD)	MHP	29.3 (7.1)	29.3 (7.1)	31.9 (7.9)	2.6 (4.3)	29.3 (7.1)	33.6 (8.6)	4.3 (5.0)
Median (IQR)	MHP	30 (24, 36)	30 (24, 36)	31 (29, 38)	3 ( 0, 4)	30 (24, 36)	36 (29, 42)	4 ( 0, 8)
Mean (SD)	no MHP	36.5 (8.3)	36.5 (8.6)	36.2 (8.4)	− 0.2 (4.7)	36.4 (8.6)	36.6 (7.9)	0.3 (5.2)
Median (IQR)	no MHP	37 (29, 43)	37 (29, 43)	36 (31, 43)	0 (− 1, 1)	37 (29, 44)	38 (30, 43)	0 (− 2, 3)
KAMH								
Mean (SD)	MHP	116.3 (15.1)	116.3 (15.1)	118.7 (15.5)	2.5 (10.6)	116.3 (15.1)	115.9 (19.0)	− 0.3 (11.7)
Median (IQR)	MHP	116 (102, 129)	116 (102, 129)	119 (101, 134)	0 (-3, 9)	116 (102, 129)	115 ( 99, 135)	− 2 (− 4, 5)
Mean (SD)	no MHP	115.6 (14.5)	115.8 (14.9)	115.8 (18.5)	− 0.0 (10.5)	115.4 (15.0)	114.6 (18.0)	− 0.8 (12.1)
Median (IQR)	no MHP	115 (106, 126)	115 (106, 126)	116 (102, 128)	0 (− 6, 3)	115 (105, 126)	115 (105, 125)	0 (− 7, 7)
Anxiety								
Mean (SD)	MHP	38.4 (10.3)	38.4 (10.3)	37.6 (11.5)	− 0.7 (8.6)	38.4 (10.3)	37.1 (11.4)	− 1.3 (9.1)
Median (IQR)	MHP	35 (31, 45)	35 (31, 45)	36 (29, 40)	1 (− 2, 5)	35 (31, 45)	33 (31, 40)	1 (− 6, 5)
Mean (SD)	no MHP	33.6 ( 7.6)	34.2 ( 7.7)	34.1 ( 8.7)	-0.1 (5.0)	34.4 ( 7.5)	34.6 ( 8.9)	0.2 (7.7)
Median (IQR)	no MHP	34 (28, 38)	34 (31, 38)	33 (30, 38)	0 (− 1, 1)	35 (31, 39)	34 (29, 40)	0 (− 4, 4)
Depression								
Mean (SD)	MHP	61.6 (19.8)	61.6 (19.8)	61.1 (16.9)	− 0.5 (12.9)	61.6 (19.8)	62.8 (16.2)	1.3 (13.9)
Median (IQR)	MHP	54 (49, 68)	54 (49, 68)	57 (50, 69)	0 (− 9, 6)	54 (49, 68)	60 (50, 73)	0 (− 9, 13)
Mean (SD)	no MHP	55.8 (15.4)	55.9 (15.6)	56.0 (14.9)	0.1 (9.4)	56.3 (15.5)	57.1 (13.5)	0.8 (12.9)
Median (IQR)	no MHP	54 (44, 64)	54 (44, 64)	52 (44, 64)	0 (− 3, 5)	55 (44, 64)	56 (45, 64)	0 (− 6, 8)

FU^*^ follow-up

### Qualitative results

Participants across all stakeholder groups demonstrated a shared interest in the game prior to engagement with the intervention. Excitement was a dominant emotion in CYP narratives which focussed on experiential and interactive elements. CYP described expectations relating to wanting an interesting and challenging intervention. This excitement appeared driven by a desire to improve mental health. Supporting quotes are provided in Additional file 1 with summary description of TFA components provided below.

Parents’ perceptions mirrored those of CYP but they were more likely to focus on education and mental health outcomes rather than experiential aspects. Facilitators also expressed a desire for a solution focused intervention which was challenging for CYP and had the potential to improve CYP abilities to communicate with parents. One CYP participant and one facilitator expressed concerns about the potential for digital games to become addictive.

### Affective attitude

Affective attitude relates to participants’ feelings about taking part in an intervention. The general consensus amongst CYP was that the game was fun, exciting and enjoyable and not overly complicated. These feelings were endorsed by parents and facilitators who described CYP as curious and eager to engage with the intervention and felt the game was well aligned to the needs of CYP. For most participants, their experiences with the intervention either met or exceeded their expectations. Parents described how they also enjoyed the game and had played together with their children on occasion. Facilitators further highlighted the uniqueness of the digital application in the Indonesian context and valued the focus on self-management and independence given parents/professional were often hesitant to talk about mental illness due to the prevailing stigma in Indonesia.

There were shared concerns raised by all three stakeholder groups about the game becoming boring after long periods or repeated engagement with the digital element of intervention. When played multiple (in excess of five) times, the game was considered to become less challenging which decreased CYP enthusiasm for the game over time. Parents highlighted rival games (non-health) that were more challenging and competed for CYP attention. Facilitators acknowledged this but appeared to better understand the intended use in the short-term and as a health intervention.

Such concerns; however, did not appear to impact on the perceived usefulness of the intervention. The positive feelings associated with the game appeared to relate to the storyline design, interactive elements, CYP’s ability to identify with the characters, the storylines reflecting real life experiences and the utility of the practical strategies which could be incorporated into their everyday life.

Suggested improvements to improve affective attitude included having more customisation, additional levels within the game, less text and more interactive elements. See Additional file 2 for more detail on suggested improvements to the prototype intervention.

## Burden

The burden component within the TFA relates to the amount of effort that is required to be invested by participants in order to engage with the intervention. Most participants across stakeholder groups did not report any burden and those were identified within this component were relatively minor.

Stakeholders across groups described initial problems downloading the game in terms of the length of time required, technical issues relating to gameplay or challenges with compatibility with their personal devices. Others described the effort to motivate themselves (CYP) or others (facilitators/parents) to play the game which was exacerbated after prolonged engagement with the intervention and when facilitation was undertaken remotely. This perceived burden appeared related to the amount of text that needed to be read within the app and the length of time required to complete chapters.

### Perceived effectiveness

Perceived effectiveness relates to participants’ perspectives about whether an intervention can achieve its intended purposes or not. Participants across all stakeholder groups described elements of the potential and actual utility of the intervention and valued the combination of group sessions and one-to-one engagement with the digital game. No participants reported any negative outcomes. Participants described the timeliness of the intervention and most reported some form of direct benefit as a result of taking part. Some felt that with further improvements to the intervention benefits could be further maximised (see Additional file 2). Figure 2 outlines the hypothesised impacts and mechanisms of change developed prior to undertaking the case study evaluation.

Whilst initially finding it difficult to articulate benefits, CYP described some positive impacts of the intervention on current mental health literacy and self-management. They also described an increased readiness to engage in self-management in the future if problems arose. These impacts appeared to be related to both increased knowledge about mental health and self-management strategies but also increased self-efficacy in terms of undertaking these activities. These included being able to control their emotions better, increased understanding about their own and others’ mental health, reduced stigma around mental health and feeling more confident to tackle problems directly. Other benefits identified by CYP included greater social confidence and improved general and mental health specific communication within families and friendship groups. Parents and facilitators supported these attributed benefits and identified additional ones which included improved mood, thinking more positively about things, increased independence, improved behaviour, enhanced problem solving abilities, less stress, being more respectful of and polite to others and being more attentive to siblings. Some felt this had been particularly useful during the pandemic because ongoing lock-downs had led to CYP becoming more introverted but others felt that the pandemic had limited opportunities to demonstrate their learning.

Benefits were also identified for facilitators, parents and families. These included better understanding of mental health and how to manage it within their families or within professional roles, improved self-management, increasing their awareness that CYP want to and can help themselves, reflecting on their parenting/professional role in relation to mental health and improved relationships with CYP. Parents and facilitators described examples of using the character and their experiences to talk to CYP about mental health and how CYP often mirrored the behaviour of characters within the intervention. A small number of parents and facilitators also described how CYP were able to challenge parents' behaviour towards their own mental health after the intervention using the characters within the digital application.

Facilitators thought the intervention might work best for those currently experiencing mental health problems and that the solution focussed nature of the game was useful as it provided insight that could be applied in daily life which increased the relevance and potential effectiveness of the intervention. Parents; however, raised the issue of long-term effectiveness when engagement with the intervention stopped. One parent retained their concern about the potential for CYP to become addicted to this type of intervention.

### Ethicality

Ethicality relates to the extent to which the values underpinning an intervention align with the participants’ own value systems. Encouragingly, the IMPeTUs intervention appeared well aligned to the values of all three stakeholder groups. Facilitators also described how the intervention responded well to recent societal shifts in Indonesia which has meant that there is greater recognition of the importance of the need to promote positive mental health and wellbeing. There were no negative aspects or outcomes identified.

Participants across groups described how children in Indonesia are familiar with engaging with digital games generally and that this was their preferred way to learn about mental health. This was perceived to increase the accessibility of the intervention in Indonesian contexts. The IMPeTUs game was compared favourably by parents and facilitators to other non-health games that CYP played because it had a more useful purpose. One parent suggested the digital game should include more religious content.

### Intervention coherence

Intervention coherence relates to the face validity of the intervention to relevant stakeholders. Generally speaking, there was good intervention coherence reported across all three stakeholder groups. CYP, parents and facilitators described the intervention to be straightforward and clear and the study documentation/processes prepared participants well for facilitating or using the intervention. This coherence appeared to be enhanced by the perception that the intervention met an unmet need for CYP in Indonesia and it was suitably different from existing interventions with similar aims.

There was acknowledgement amongst facilitators that there needed to be sufficient time built into the implementation process in order to achieve intervention coherence amongst parents and CYP. This was considered more difficult to achieve when facilitation was undertaken remotely due to Covid-19 related restrictions. Introducing the game and clarifying understanding amongst study participants was considered easier to achieve if undertaken face-to-face.

Some facilitators felt that coherence was more readily achieved amongst CYP and required more sustained effort to achieve in parents. This appeared to be due to parental concerns about CYP engagement with digital games generally, a lack of understanding about how a game could improve mental health amongst parents and concerns about digital literacy amongst some adults when compared to children.

Facilitators reported this could be mitigated against through having regular meetings/contact with parents and all participants having the opportunity to ask questions and raise concerns. This worked best where facilitators took responsibility for driving these activities.

One CYP reported finding the storyline and sentences confusing and a small number of parents endorsed this whilst highlighting that understanding was more of an issue for younger participants. This was a view that was also acknowledged by some facilitators.

### Opportunity costs

Opportunity costs relate to the benefits, profits or values that need to be relinquished in order to engage with an intervention. Participants across all three stakeholder groups coalesced in their views that there were no identifiable opportunity costs related to engagement with the IMPeTUS interventions. One parent reported that the cost-free access to the current intervention was a comparative strength compared to other pay to play digital applications.

### Self-efficacy

Self-efficacy is defined at the participants' confidence that they can perform the behaviours required to engage in the intervention. Self-efficacy was not reported as a major component of intervention acceptability in the parent data, most likely reflecting their more peripheral role as a support rather than direct participant in the intervention.

Facilitators received training to deliver the IMPeTUS intervention and considered this training fit for purpose. They felt that the intervention aligned well with their existing roles, and that their previous experience and existing skills had prepared them well. A minority of facilitators suggested increasing the level of training to ease any necessary transition from face to face to remote facilitation. Some also acknowledged that competing occupational responsibilities and time pressures had impacted their ability to fully familiarise themselves with the app, and advised that sufficient opportunity should be available to enable facilitators to engage with the intervention themselves before introducing it to CYP.

Despite facilitators requiring time to orientate themselves to the game, and explain it to parents, they believed that the digital IMPeTUS intervention was intuitive for CYP and required little preparation. This view was upheld by the CYP themselves; some of whom explicitly acknowledged that they were able to engage in the impetus intervention during periods of low mood, when participation in other gaming applications was more difficult.

Distinguishing between the IMPeTUS app and the group facilitation components of the intervention, some CYP reported that, due to a lack of confidence, they initially found the group discussions challenging. However, there was no evidence that these feelings impacted on CYP’s participation and group discussion was still considered a beneficial part of the intervention. Group discussion was reported to increase CYP’s ability to initiate and engage with the game, as well as increasing their mental health knowledge and understanding.

## Discussion

In line with WHO guidelines for evaluating prototype mHealth interventions, our mixed methods case study evaluation aimed to examine the usability, feasibility and preliminary impact of our co-produced intervention [33]. We recruited 56 CYP, 49 parents and 18 facilitators to understand user perspectives on these criteria and underpinned our study with a co-produced theory of change to ensure a theory-driven evaluation.

The data analysis revealed that usability was strongly impacted by the perceived function of the intervention and whether its primary function was considered to be an interactive game or a mental health intervention. Our qualitative analysis indicates that our intervention fits best in the mental health intervention landscape. In this context, our intervention has an explicit purpose (mental health education and self-management), and thus a potentially targeted and time-bounded duration of use, partially offsetting user concerns about longer term use and gaming sustainability more critical to a digital entertainment context. While gaming innovations may help to maximise the uptake and interactivity of digital health resources, further consideration may need to be given to the development and evaluation of products capable of performing well in both markets.

Through consultation with our PPI advisory group and study team, we envisaged that participants would need to engage for a minimum of one hour in each storyline chapter of our intervention to confer benefit in line with our co-produced theory of change. However, some participants reported using the intervention primarily as a recreational game and played it multiple times over sustained periods of time. This unintended usage could lead to participants reporting repetition and/or boredom and highlights a need for clear explanation of the aims of the intervention and expected engagement.

Engagement in digital health interventions is likely to be a function of both the intervention and user characteristics. Participants across stakeholder groups felt that there was potential for further intervention development, through the addition of extra chapters and improvements to existing content, to increase future sustainability (See Additional file 2). Examples for additional chapters included self-harm and suicidal ideation, bullying, and  substance abuse. There were also some indications that in its existing form, our intervention might work best for: older children, those with less gaming experience, those currently experiencing mental health problems and those with very limited existing mental health literacy.

Our data identified good levels of acceptability with our intervention across stakeholder groups. Using a checklist of design features which are known to enhance user experience, our intervention performed well terms of interface aesthetic, personalisation, navigation, reinforcement and message presentation [40]. There were no concerns reported about credibility which was considered to be as a result of the implementation route (via a trusted organisation) but participants described a preference for enhanced communication within the game (e.g. with others using the app) in future iterations [40]. Encouragingly, there were minimal burdens identified and no negative outcomes reported. This is likely to be a consequence of our iterative co-productive design approach which drew on principles of experience-based co-design with no a priori restrictions in terms of intervention content or delivery [30]. Our substantial needs assessment in the form of two systematic reviews [27, 28] and qualitative research with CYP, parents and health and education professionals [31, 32] was used to inform initial co-design workshops with the games designers and CYP, parents and professionals who together agreed upon a brief for our prototype intervention [26]. Games designers and researchers then worked closely with patient and public involvement contributors to implement the brief with two opportunities for initial user testing and iteration. This appears to have contributed to the perceived timeliness and relevance of the intervention and the close alignment of the intervention with CYP needs, values and real word experiences [41]. It also likely reflects a wider social shift in Indonesia from preferences for traditional practitioners to evidence-based interventions and alternative forms of service provision [42]. Our analysis demonstrates stakeholder preferences for mHealth interventions which focus on empowerment and recovery (i.e. solution-focussed) interventions rather than paternalistic problem-focussed support and resources. This highlights the opportunity for harnessing the drive for global movements that prioritise self-management and strengths-based initiatives, for the development of mental health interventions in Indonesia [43].

Our qualitative data identified some initial concerns about the addictiveness of computer games and digital applications more generally which have been identified in Indonesian contexts previously [26]. These concerns appeared to be allayed for the most part by direct experience with our intervention; we identified some important attitudinal shifts which appeared to be driven by engagement with the intervention and the range of benefits identified.

Our preliminary theory of change which was co-produced with relevant stakeholders during the initial co-design process identified a range of theorised benefits [26]. However, our qualitative data identified a more nuanced range of benefits that were not adequately captured in our original theory of change or quantitative outcome measures. These included improved conceptualisations of mental health and readiness to disclose difficulties, increased confidence to communicate with others and increased likelihood of engaging in positive mental health self-management. Our quantitative outcome measures focussed on current mental health symptoms/difficulties which may not have been sufficiently relevant to our participants (of whom only 19/78 were currently experiencing mental health difficulties) or sensitive to capture these more nuanced benefits.

Our quantitative data tentatively supported the qualitative data by indicating an increased utility of intervention for those currently experiencing mental health problems. These CYP (n = 19) had lower quality of life scores, lower family adaptability and cohesion and higher anxiety and depression scores at all three assessment points compared to children who did not report experiencing a mental health problem. There was some evidence that these children exhibited a greater change from baseline, particularly in quality of life and family adaptability and cohesion (both of which improved). However, only 19 CYP reported experiencing a mental health problem, so these findings must be viewed cautiously until data from a much larger sample is obtained.

The qualitative data also identified a range of spill over impacts which were unanticipated at study outset. For example, parents and facilitators reported benefiting directly from their experience with the intervention but also acknowledged wider family benefits, not incorporated in our initial theory of change. These benefits included CYP helping parents with their own mental health, enhanced understanding of and empathy towards the challenges CYP currently face and increased mental health literacy amongst parents, families and facilitators. There has been a step change towards increasing the availability of lower intensity, scalable interventions for depression and anxiety globally [44]. However, in the current study facilitators described limited awareness of the use of mhealth interventions to support self-management for mild-moderate mental health difficulties prior to using the intervention.

**Fig. 4:**
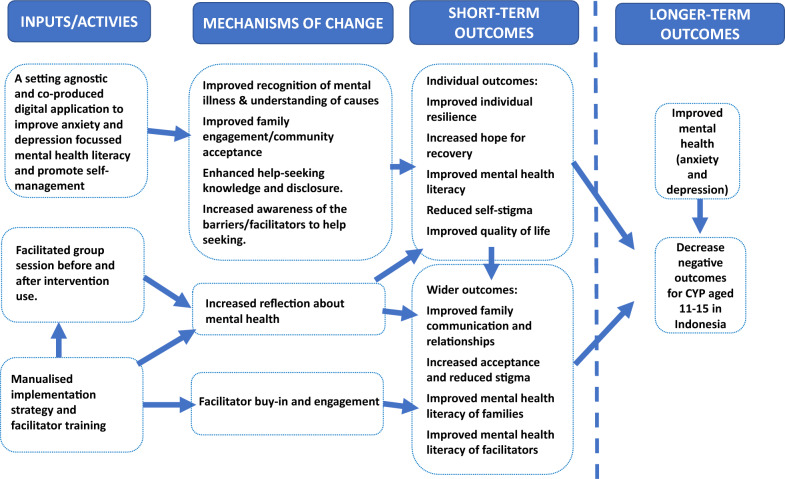
Revised Theory of Change

WHO guidelines for evaluating interventions in the later stages of the intervention maturity life-cycle recommend evaluation in terms of effectiveness, cost-effectiveness and implementation [33]. While our findings are limited in their capacity to directly evidence population health change, they have important implications for future evaluation. Revising the intervention’s theory of change and ensuring the selection of meaningful quantitative measures and the recruitment and inclusion of potential beneficiaries are important activities in future evaluation processes. Public involvement in the design of future evaluations will further support the choice of relevant and appropriate outcome measures and evaluation processes. Future implementation will also require effective collaboration with relevant ministries and institutions in Indonesia such as the Ministry of Health, the Ministry of National Education and the Ministry of Communication and Informatics. See Fig. 4 for a revised theory of change.

Initial findings from our prototype evaluation highlight the value of the use of our digital intervention in conjunction with the pre-and post group sessions despite these sessions being initially viewed as challenging by CYP in both on and offline settings. These appeared particularly important in the implementation of the intervention in Indonesian contexts given the concerns parents raised about not feeling comfortable discussing mental health with their children and because they offset the increased curiousness as reported by CYP [31]. There were no differences in study data between different implementation sites (schools, health services and community venues or geographical locations) suggesting our intervention is setting agnostic and can be used on or offline. However, the data highlighted the importance of facilitation and of recruiting facilitators from existing contexts with pre-existing positive relationships with CYP. Facilitators also suggested developing supporting training materials such as information videos and manuals to further support future implementation. Our mixed methods case study evaluation thus provides important learning for definitive clinical and cost-effectiveness studies and opportunities for optimising future implementation strategies.

### Limitations

Our mixed methods study recruited from three study sites in Java, Indonesia (Jakarta, Bogor and Magelang). Indonesia has high levels of geographical, economic and cultural diversity and as such, our findings might not be transferable to other areas of Indonesia. Participants in the current study self-selected to participate in the study. It may be that those with an interest in mental health and digital interventions were more likely to volunteer to take part. Future research is required to evaluate our intervention with other groups including those with lower levels of digital literacy.

Our quantitative data collection processes were not powered to detect statistical difference and the aim was instead to assess the feasibility of the future evaluation of our intervention and interrogate our theory of change. While completion levels indicate high levels of feasibility in terms of future evaluation, robust randomised controlled trials are required to adequately assess clinical and cost effectiveness. The largest absolute changes are in the PedSQL at one month (+ 2.5 points). A clinically important change for a quality-of-life measure is considered to be in the region of 5 points. For example, if such data were used to inform a power calculation for a subsequent RCT to test the effectiveness of the game (versus, say, usual care), it would likely result in a very large sample size being required.

## Conclusion

Our co-produced digital anxiety and depression focussed mental health literacy and self-management intervention was considered acceptable and easily implementable by CYP, parents and facilitators even in the context of the Covid-19 pandemic. The intervention is currently being culturally adapted for CYP in the UK demonstrating its flexibility for use in high and low resource settings. The chapter focussed design easily allows for expansion of our intervention which can be developed specifically for the needs of local populations, maximising its relevance and scope to include additional conditions, including comorbid non-communicable diseases and integrated care. Future research should explore equity in access to digital interventions in Indonesia and consider the impact of the digital literacy on engagement with our intervention.

Digital applications to support mental health literacy and self-management are potentially an acceptable and feasible way in which health, community and education settings can attempt to prevent future, and treat existing, burdens of common mental health problems amongst CYP in Indonesia. The results will be used to further refine our co-produced intervention prior to definitive evaluation of its clinical and cost effectiveness.

## Supplementary Information

**Additional file 1: **Example quotes.

**Additional file 2: **Suggestions for future iterations of the digital application.

## Data Availability

The datasets generated and/or analysed during the current study are not publicly available due to ethical restrictions but are available from the corresponding author on reasonable request.

## Abbreviations

CYP: Children and young people
EBCD: Experience based co-design
LMIC: Low and middle income country
MHL: Mental health literacy
MHP: Mental health problems
PPI: Patient and public involvement
ToC: Theory of change
TFA: Theoretical framework of acceptability
UK: United Kingdom
