# Supplementary material for: Evaluating a prototype digital mental health literacy intervention for children and young people aged 11–15 in Java, Indonesia: a mixed methods, multi-site case study evaluation
Source: Child Adolesc Psychiatry Ment Health. 2023 Jun 26;17:79. doi: 10.1186/s13034-023-00608-9 (PMC10294420; doi:10.1186/s13034-023-00608-9)
Supplement: Supplementary file 1 — Additional file 1: Example quotes. [file 13034_2023_608_MOESM1_ESM.docx]

Supplementary File 1: Example quotes for qualitative data analysis by TFA component

| **TFA Component** | **Supporting quotes** |
| --- | --- |
| Affective attitude | *"It was entertaining... It was fun... It wasn’t too complex".* **Site 1, Focus Group, CYP: ID1110**  *They’ve been enjoying it so far. “I like the game, Mom.” They feel that the game is challenging.* **Site 5, Focus Group, Parent: ID5203.**  *Seeing from the results of the game, I think that it did meet my expectations. It’s quite interesting, the pictures and text are easily digestible. I think it’s a good starting point for mental health education for kids. [] And personally I don’t think it’s boring because the game itself doesn’t feel childish, so it suits young people just fine, with the text and all. [] I think the format is quite good already, and it has a good structure: the background, then the case itself, then the next story.* **Site 1, Interview, Facilitator: ID1303.**  *It’s fun, but after playing it for a while, I got bored.* **Site 9, Focus Group, CYP: ID9104**  *At first [my child] was playing the game happily. But after a few months, they said, “I’m finished, Mom.” Like, they said that there was no more variation in the game, so they were pretty much done. After a while, they’d just open the game once a week or something. But at first, they liked it a lot. They said that it was clever, err, interesting. They said that they liked customizing the character, but after a while, they said that they’d finished all the levels. The game was complete, so they only opened it occasionally after that.* **Site 1, Focus Group, Parent, ID: 1208**  *Yes, it absolutely met my expectations. It even blew my expectations out of the water, because the game was very exciting. I understand that it’s not as attractive as other games, because it’s an educational game, but it still did meet my expectations.* **Site 6, Interview, Facilitator, ID: 6301**  *The most useful thing was finding problems in real life that are similar to the ones in game... Although I didn’t find everything. The game made me feel more refreshed... A weight off my shoulder... Because I learned that I can handle my problems.* **Site 8, Interview, CYP: ID8101** |
| Burden | *My impression was that delivering it wasn’t very hard, because well... I work in a hospital, and [the kids] who are involved are ones who have been treated here. There weren’t a lot of problems, and the kids received it well. They welcomed the idea. They were even asking a lot of questions; they were very enthusiastic. They kept asking, “How do we play the game?” So I think I had a good impression, and the received it quite well.* **Site 1, Interview, Facilitator: ID1303.**  *Sometimes I felt bored because it was all just songs and text... I’m too lazy to read through them... It’d be more exciting if there were voices... If the text was voiced... If the text were read to me.* **Site 1, Focus Group, CYP: ID1108**  *…because my child told me that sometimes, in the middle of playing, the game would suddenly just stop.* **Site 8, Interview, Parent, ID8203**  *Communicating with the children. sometimes we’d ask a question via WhatsApp in the afternoon, but we’d only receive a reply in the evening. We were hoping that we would get answers as soon as possible.That’s all we did... And well, we also encouraged them to play after that.* **Site 8, Focus Group, Facilitator, ID8302.** |
| Perceived effectiveness | *People here aren’t very aware of mental health just yet. I’m sure that they would benefit from it, but you need to make sure they’d be interested enough to download the game, and you have to do that by improving the game itself.* **Site 8, Interview, Parent, ID: 8201**  *5104: it’s very helpful. I learned that I have to talk and not hide it when I have problems, because otherwise I might get stressed.*  *5104: The important thing was that the game taught me more about myself.*  *5106: They will benefit from this, because this game is very educational. There will be benefits for playing this with friends your age, because this game teaches you about mental disorders and how to handle them.*  *5108: The benefit is that [they will know] that when they’re anxious, they shouldn’t conceal it, because if they do that, their mental [health] would take a hit, and their anxiety would get worse.* **Site 5, Focus Group, CYP**  *1208: I feel that [my child] enjoyed it, and felt that it benefited them, because they once said, “Mom, in Impetus, something something, I have anxiety, so I have to be able to control myself.” So, [my child[ previously didn’t know what anxiety was, how to look after themself, how to distract themself, what to do when they’re anxious... Now you can see that they changed a lot, and they’re happy. I think it’s because they’ve played the Impetus game. [When I was asked to participate[ I was so happy, because I’ve brought [my child] to consult a psychologist before, and we even rented some kind of kit bag, because whenever [my child] saw the rain, they would be very hysterical hearing all the thunders. They’d just jump and keep crying. Astaghfirullah al azim—I seek forgiveness in God the Almighty—they were almost... But after they found Impetus, they started to be able to control themself.* **Site 1, Focus Group, Parent: ID1208.**  *When I found out about the game, I hoped that it would give a new nuance to the kids... a game that would be educational to them. So the kids wouldn’t only enjoy playing the game, but they would also learn educational things from it. Alhamdulillah—praise be to God—in our last Zoom session, there were a lot of positive responses from the parents. They said that their kids changed after playing the game........The benefits for me is that I now know there are educational games as well, not only games that you only play for fun. As for the kids, the parents said that they saw changes in their kids’ behavior.* **Site 3, Interview, Facilitator, ID: 3033**  *I would remind them about the game, how the character handled it, what to do to deal with the problem.* **Site 7, Focus Group, Parent: ID7208.**  *Moreover, one of them actually reminded their mom about emotions and such, like “Mom, if you’re angry, this is what you have to do...”.* **Site 7, Focus Group, Facilitator: ID: 7303.**  *There are a lot of benefits to this game. A lot of them... Like now, I can understand that a game about mental health exists. That I can learn about mental health from the game. And I learned about things... I learned that there are other types of help other than therapy... Like online therapy, such as this game. For your own mental health... And I could use this game as my online therapy as well.* **Site 6, Interview, Facilitator, ID:6302** |
| Ethicality | *Moreover, now there are a lot of shows on social media or on TV with very dangerous content, and those shows are just ignored [by society]. That’s the exact same thing as mental illnesses. That’s why we must work together with the parents and the family, also academics and professionals, Muslim scholars, prominent figures in society, and religious figures... We all have to continuously... This isn’t just the responsibility of health professionals, but it’s the responsibility of all of us.* **Site 3, Interview, Facilitator: ID3303**  *I didn’t think anything, I just thought, it’s a game, so I would play it.* **Site 5, Focus Group, CYP: ID5104.**  *It turns out that, learning from games is... Hmm. It turns out that we can share our knowledge about mental health, and I saw that the kids weren’t really aware that they were learning. I imagine they wouldn’t be very enthused if they had to read books to learn something, but if they’re playing a game, they wouldn’t feel that they’re actually learning new information. I think that’s it.* **Site 1, Interview, Facilitator: ID1303.**  *Everything has to come back to ruqyah healing. Everything will come back to The Creator Of All, so I hope that the game would have sentences that have something about the ruqyah healing in it, so that children understand that everything will come back to Allah, and that they wouldn’t forget about Allah even when playing the game.* **Site 6, Focus Group, Parents: ID:6210** |
| Intervention coherence | *It was easy, because it was simple* **CYP Interview 8101**  *I think that it [training] was quite adequate and quite good. We understood what we should do. That’s how I feel.* **Facilitator interview 1301**  *Previously, to improve literacy, people normally would need to read books, hard copies, or cards, things like that. In this digital era, and in this pandemic, an application or a game like this is very suitable for the current condition.* **Interview, Site 1, Facilitator: ID1302**  *I delivered the Impetus game by inviting the parents to introduce it to them, so that not only the children, but the parents understand it as well.......For me, it’s communicating with the parents first so they’d understand about it. The children understood really fast... You only had to tell them once, and then they’d understand quickly.* **Interview, Site 3: Facilitator: ID3301**  *Because this is a game, kids would understand it better than us who are older. Older people aren’t as technologically savvy as kids.* **Interview, Site 3: Facilitator ID: 3302.**  *We met up a few times with the children and the parents via Zoom. As a teacher, I think that it was very good, and the parents then knew that the game was good for their children. I think there should be regular meetings in the future, perhaps an evaluation session every month, so that the improvement can be continuous and not just stopping at one point.* **Facilitator interview, Site 3, 3303.**  *I didn’t really understand. I didn’t really get the solutions, and it was kind of confusing*. **Focus Group, Site 9, CYP: ID: 9101**  *It is not yet appropriate for their age, they are still very young. A child only thinks about playing*. **Focus Group, Site 2, Parent: ID: 2204**  *11-year-olds tend not to understand when we initially explained it to them, they don’t know about mental health yet... When we explained the game to them, it seemed that they were a bit... apprehensive about it.* **Interview, Site 2, Facilitator: ID2301.** |
| Opportunity costs | None reported. |
| Self-efficacy | *I was prepared to deliver the game. More initial training for facilitators might have been helpful to improve confidence: Maybe you can take longer in the beginning so people can play the game together. Or maybe you could make a presentation to introduce this game, so that it’d be easier for us to explain it to the students.* **Interview, Site 6, Facilitator, ID6302.**  *I think the children are smarter than us. When I tried playing the game, the children were the ones who taught me how to do it. They only had to take a glimpse at it, and they already know what to press and such.* **Focus Group, Site 7, Facilitator, ID7304.** |
