# Supplementary material for: Evaluating a prototype digital mental health literacy intervention for children and young people aged 11–15 in Java, Indonesia: a mixed methods, multi-site case study evaluation
Source: Child Adolesc Psychiatry Ment Health. 2023 Jun 26;17:79. doi: 10.1186/s13034-023-00608-9 (PMC10294420; doi:10.1186/s13034-023-00608-9)
Supplement: Supplementary file 2 — Additional file 2: Suggestions for future iterations of the digital application. [file 13034_2023_608_MOESM2_ESM.docx]

Supplementary File 2: Suggestion for future versions of the IMPeTUs application.

|  | **What needs to be modified** | **Why does it need to be modified** | **How could it be modified** |  |
| --- | --- | --- | --- | --- |
| **Children and young people** | Number of players |  | Include multiplayer options |  |
|  | Game content | Too much text to read | Adding audio (voiceover) to provide information; reduce the text to read, have shorter sentences |  |
|  |  |  | Add more content about mental health (i.e. theory, explanations, how to control emotions) |  |
|  |  | After multiple plays the game became repetitive/boring | More chapters with more complex and less predictable storylines; introduce levels to game; include more and more challenging minigames; add option to save progress |  |
|  | Visuals |  | Brighter colours; introduce different settings (e.g. outside the school and home); more characters; more customisation for characters (e.g. clothing); add more about controlling emotions; moving animations (e.g. 3D, move your own character, character expressions) |  |
|  | Software |  | Add a back button; add the game to the 'Playstore' |  |
|  |  |  | Describe more attractively on the 'Playstore'; improve the icon |  |
| **Parents** | Visuals | To be more appealing, interesting | Brighter colours; introduce moving animations; more characters |  |
|  | Game content | To reduce boredom and repetitiveness; make more interesting and challenging; to compete with other games on the market | More customisation; more updates and continuations of the game; more and more challenging, varied storylines (e.g more than two options to questions); introduce levels to game (e.g. the possibility to fail levels); more rewards |  |
|  |  | Too much text to read; so don't have to read text | Adding audio (voiceover) to provide information; reduce the text to read, have shorter sentences; use simpler, more young person friendly language |  |
|  |  | It was felt the chapters end on a 'cliffhanger' | Give more of a solid change to the main character at the end of the storyline |  |
|  |  | Concern about the lack of religious content in the game | Add content around the religious issue in dealing with mental health problems |  |
|  |  |  | (Consider adjustment of game in line with governmental programmes- i.e. Ministry of Education and Culture's 4 C's) |  |
|  |  | To take into account gender, age and 'maturity' of young person; to reduce boredom | Customise game depending on age and gender (e.g. visuals, amount of text) |  |
|  | Background music |  | More music; make the music less soft |  |
|  | Target audience | So all young people are able to play | Increase the target range for the game |  |
|  | Software |  | Ensure that the game plays smoothly (e.g. does not stop in the middle) |  |
| Professionals | Facilitator training | To help improve explanation of the intervention to parents; to help facilitators gain a better understanding of the intervention (e.g. to give better help to young people); to help those faciliators less tech-savvy | Giving faciliators more information about the research (e.g. purpose) and game content; use simpler wording; allow more time for training (e.g. so faciliators can help young people download app and they can try out the app together); better explain the reward system in the game; more information on those devices that the intervention can and cannot be downloaded on; access to the intervention prior to training; more information of how to download app (i.e. tutorial); more resources (i.e. presentation) to help introduce the app to the young people |  |
|  | Visuals | To be more appealing | Introduce different settings outside the school and home (e.g. park, mall, café); introduce moving animations; have characters that kids 'like'; characters with unique appearances, more eye catching |  |
|  | Background music |  | Make the music more encouraging and cheerful; mellow |  |
|  | Facilitator role and support | Need better support to address app compatibility/access issues |  |  |
|  |  | To better facilitate the delivery of the intervention | More regular check ins with professionals |  |
|  |  | Interacting online made it more difficult to help young person; face-to-face would give more contact time with young person | Face-to-face meeting preferred for introducing the intervention and the group sessions |  |
|  | Game content | To make more interesting; make it more challenging | More updates and continuations of the game; make the game shorter; include more chapters (e.g. additional mental health conditions to anxiety and depression); include more varied, relatable storylines (e.g. school or friend problems); introduce levels; more rewards (e.g. points, applause); more interactive game and mini games; more minigames; more input from young people about what they want in the game |  |
|  |  | Too much text; intervention had a lot of medical words that needed explaining to young person and parent | Adding audio (voiceovers); reduce the text; use simpler, more young person friendly language; bigger text; easier to understand instructions |  |
|  |  | To add content to the existing game | Add content around the religious issue in dealing with mental health problems |  |
|  |  |  |  |  |
|  |  |  | Add practical methods of looking after mental health (e.g. how to overcome angry and insecure feelings) |  |
|  |  |  | Add information about mental health resources in the local area |  |
|  |  |  | Add a 'basic psychology test' at the beginning and have the storyline develop from the results |  |
|  |  | Take into account age | Customise game depending on age (e.g. more colourful for younger people) |  |
|  | Evaluation of the intervention | Too much time between sessions | Shorten the evaluation period |  |
|  |  | So improvements can be made continuously rather than at one timepoint | More evaluation sessions (i.e. an evaluation session every month) |  |
|  | Software | Intervention needs to be easier to install on devices; mobile devices have limited memory to download intervention | Add the intervention to the 'Playstore' (i.e. so 'application' is not as big); allow for intervention to be downloaded onto laptops; access to internet (data) to download intervention |  |
|  | Number of players |  | Include multiplayer options |  |
|  | Roll out |  | Consider using game in other settings (e.g. hospitals, community health centres) |  |
|  |  |  | Consider marketing game to gain interest |  |
